# Exercise adherence and wrist function recovery after distal radius fracture surgery in older adults: a 12 week prospective longitudinal study

**DOI:** 10.3389/fmed.2026.1907971

**Published:** 2026-08-28

**Authors:** Sunan Ji, Yiping Sun, Yu Zhu

**Affiliations:** 1 Department of Orthopedics, The Sixth People's Hospital of Nantong, Nantong, Jiangsu, China; 2 Department of Outpatient and Emergency Operating Room, Affiliated Hospital of Nantong University, Nantong, Jiangsu, China

**Keywords:** distal radius fracture, exercise adherence, functional recovery, interaction effect, longitudinal study, older adults

## Abstract

**Objective:**

To explore the interactive relationship between wrist function recovery trajectory and exercise adherence in older patients after surgical treatment of distal radius fractures.

**Methods:**

This study was a prospective, multicenter, observational study. A total of 230 older patients with distal radius fractures who underwent volar locking plate internal fixation at Nantong Sixth People's Hospital and Affiliated Hospital of Nantong University from February 2025 to February 2026 were enrolled. Wrist function was assessed using the Patient-Rated Wrist Evaluation (PRWE) at 2, 4, 8, and 12 weeks postoperatively. Exercise adherence was evaluated using the Orthopedic Patient Functional Exercise Adherence Scale. Wrist range of motion and grip strength were also measured. A linear mixed-effects model was used to analyze the functional recovery trajectory and the interaction effect between time and exercise adherence, with adjustment for potential confounders including age, sex, AO fracture classification, dominant hand injury, and baseline kinesiophobia level.

**Results:**

The total PRWE score decreased from 68.5 ± 12.3 at 2 weeks postoperatively to 19.3 ± 9.7 at 12 weeks. The total exercise adherence score showed an initial increase followed by a decline, peaking at 4 weeks (61.8 ± 7.9). The linear mixed-effects model revealed significant main effects of time (β = −6.82, *P* < 0.001) and exercise adherence (β = −0.53, *P* < 0.001), as well as a significant interaction between time and adherence (β = −0.04, *P* = 0.043), indicating that patients with higher adherence exhibited faster functional recovery. Convergent validity analysis demonstrated moderate to strong correlations between PRWE and objective measures (*r* = −0.39 to −0.71).

**Conclusion:**

Exercise adherence was significantly associated with the functional recovery trajectory in older patients after distal radius fracture surgery, with higher adherence being related to a more favorable functional recovery trajectory. Clinically, attention should be paid to maintaining adherence during the 4- to 8-week postoperative period, and targeted interventions should be provided for patients with high levels of kinesiophobia.

## 1 Introduction

Distal radius fracture (DRF) is one of the most common clinical fractures, with a distinct age-related epidemiological pattern. In younger individuals, these fractures are typically caused by high-energy trauma such as motor vehicle accidents, falls from height, or sports injuries. Among people aged 60 years and older, however, the incidence increases markedly, with falls being the predominant cause; DRF often serves as an important sentinel event for osteoporotic fragility fractures (1, 2). China is undergoing rapid demographic transition, with its population aged 60 years and above already exceeding 250 million and continuing to grow (3). Against this backdrop, geriatric distal radius fracture has evolved from a purely orthopedic problem into a public health challenge closely linked to aging, bone loss, increased fall risk, and declining functional independence (4, 5).

For older patients, the goal of treatment extends far beyond anatomical reduction and bony union; the core objective is to maximize recovery of wrist function and activities of daily living, thereby preventing a vicious cycle of functional decline and disability triggered by a single fall-related fracture (6, 7). Surgical intervention (e.g., volar locking plate fixation) provides stable mechanical conditions for early mobilization, yet the ultimate rehabilitation outcome depends heavily on systematic and sustained postoperative exercise (8). However, postoperative rehabilitation in the older population faces multiple unique challenges, including underlying osteoporosis, higher comorbidity burden, cognitive changes, and—critically—fear-avoidance beliefs and kinesiophobia (9, 10). Many older patients harbor strong fears about pain and the possibility that movement may lead to re-injury; this psychological state can significantly undermine their confidence in and adherence to rehabilitation exercises (11, 12).

Exercise adherence is a key modifiable factor determining orthopedic rehabilitation outcomes (13). Currently, one of the most sensitive and specific patient-reported outcome instruments for assessing wrist function is the Patient-Rated Wrist Evaluation (PRWE), which comprehensively quantifies pain and functional disability (14). Although its value has been widely recognized, existing studies have notable methodological limitations. On one hand, most studies have adopted cross-sectional designs, which can only evaluate the relationship between adherence and function at a single time point, failing to capture their dynamic association over time (15, 16). On the other hand, even among the few studies that conducted repeated follow-ups, adherence was often treated as a fixed baseline characteristic, or repeated measures analysis of variance was used merely to compare endpoint differences between adherence groups, without examining the interaction effect between adherence and time on the rate of functional recovery (15, 16). This research paradigm has clear limitations: it can neither delineate the dynamic trajectory of early postoperative functional recovery nor reveal the potentially bidirectional or interactive relationship between exercise adherence and functional recovery that may evolve over time. For example, early functional improvement may enhance exercise confidence, thereby increasing subsequent adherence; conversely, early pain and dysfunction may lead to fear and avoidance, forming a vicious cycle. Moreover, the factors influencing exercise adherence in older patients are complex and multifaceted. Previous studies have identified potential factors across multiple levels, including individual-level factors (age, sex, education, health literacy, pain, depression, frailty, cognition), psychological factors (kinesiophobia, self-efficacy, rehabilitation beliefs), social factors (family support, socioeconomic status, access to rehabilitation), and disease-related factors (fracture type, comorbidities, postoperative complications) (17–19). However, existing literature has yet to provide a comprehensive examination of these factors, and in particular, there is a lack of systematic analyses that place kinesiophobia alongside other psychosocial factors within the same framework. Therefore, to address this evidence gap, the present study adopts a 12-week longitudinal design focusing on patients aged 60 years and older who have undergone surgery for distal radius fracture. We repeatedly assess wrist function and structured exercise adherence at four critical time points: 2, 4, 8, and 12 weeks postoperatively. The primary objectives are: first, to delineate the dynamic trajectory of postoperative wrist function recovery in older patients; second, to explore the interactive relationship between exercise adherence and the functional recovery trajectory, specifically how adherence level influences recovery speed and how immediate functional status reciprocally affects subsequent adherence behavior. The findings are expected to provide key evidence for establishing evidence-based, timely, and individualized rehabilitation intervention strategies for older fracture patients.

## 2 Subjects and methods

### 2.1 Study subjects

This study is a prospective, multicenter, observational longitudinal cohort study with a 12-week follow-up period. Data were collected at Nantong Sixth People's Hospital and Affiliated Hospital of Nantong University from February 2024 to February 2026. (1) Inclusion criteria: ① Age ≥ 60 years; ② Confirmed diagnosis of acute, unilateral, closed distal radius fracture (AO/OTA type 23) by X-ray or computed tomography, treated with volar locking plate internal fixation; ③ Alert and oriented, with basic reading comprehension ability sufficient to complete questionnaires independently or with researcher assistance; ④ Stable residence, voluntary participation, and commitment to completing all follow-up assessments through 12 weeks postoperatively. (2) Exclusion criteria: ① Open fracture, pathological fracture, or concomitant ipsilateral upper extremity injury requiring activity restriction; ② Pre-existing severe osteoarthritis, deformity, neuropathy, or prior surgery involving the affected wrist, resulting in abnormal baseline functional status; ③ Severe cognitive impairment, psychiatric disorder, or uncontrolled systemic serious disease expected to preclude rehabilitation cooperation; ④ History of substance or alcohol abuse.

Sample size calculation was performed using GPower 3.1 software based on the framework of a linear mixed-effects model (repeated measures) (20). The core objective was to test the interaction effect between “time” and “exercise adherence” on wrist function. Parameters were set as follows: effect size (Cohen's f^2^) = 0.10 (small to medium), significance level (α) = 0.05, statistical power (1 – β) = 0.80, four measurement time points, and intraclass correlation coefficient = 0.50. The calculated minimum required sample size was 92 participants. Considering potential attrition in longitudinal studies, a dropout rate of 20% was assumed based on similar studies (8). To ensure an adequate effective sample size and to provide stability for the complex model estimation, the present study planned to expand the recruitment target to a final sample size of 230 participants. A *post-hoc* power analysis indicated that, given the current sample size (*n* = 230) and the observed interaction effect size, the actual statistical power was approximately 0.89, which is sufficient to reliably detect the interaction effect. This study was approved by the Ethics Committee of Nantong Sixth People's Hospital (Approval No.: NTLYLL2024033).

### 2.2 Study instruments

(1) General information questionnaire

This questionnaire collected data on age, sex, body mass index, dominant hand injury status, AO/OTA fracture classification, and kinesiophobia level. Kinesiophobia was assessed using the Chinese version of the Tampa Scale of Kinesiophobia (TSK-11) (21). The scale consists of 11 items, with a total score ranging from 11 to 44; higher scores indicate greater fear of movement and re-injury.

(2) Patient-rated wrist evaluation (PRWE)

Wrist function recovery was assessed using the PRWE, developed by MacDermid et al. (22) in 1998. It is currently the most widely accepted wrist-specific patient-reported outcome measure in the field of distal radius fractures, quantifying both pain and limitations in daily function. The present study used the Chinese version of the PRWE, which was culturally adapted and validated by Xu (23). The scale comprises 15 items: the Pain subscale includes 5 items (pain at rest, pain with repeated movement, pain with heavy lifting, worst pain, and pain frequency), and the Function subscale includes 10 items (6 specific activities and 4 daily activities). For scoring, the scores of the 5 pain items are summed directly to obtain the pain dimension score (range 0–50); the scores of the 10 function items are summed and divided by 2 to obtain the function dimension score (range 0–50). The total PRWE score is the sum of the pain and function scores, ranging from 0 to 100, with higher scores indicating more severe wrist pain and functional impairment. In the pilot test of the present study, the Cronbach's α of the Chinese version of the PRWE was 0.91, indicating good internal consistency.

(3) Exercise adherence

Postoperative exercise adherence was assessed using the Orthopedic Patient Functional Exercise Adherence Scale, developed and validated by Tan et al. (24). This scale is a self-report instrument specifically designed for orthopedic patients, with good content validity and cultural applicability, particularly suitable for evaluating functional exercise behaviors in home rehabilitation settings. The scale consists of 15 items organized into three dimensions: physical performance adherence (8 items), psychological persistence adherence (4 items), and active learning adherence (3 items). All items are rated on a 5-point Likert scale, ranging from 1 (“completely unable to do”) to 5 (“completely able to do”). The total score is the sum of all item scores, ranging from 15 to 75, with higher scores indicating higher levels of exercise adherence. To reduce recall bias and social desirability bias inherent in self-reported data, a simple home exercise diary was used as a supplementary cross-validation tool. Patients or their primary caregivers were asked to briefly record daily whether exercises were completed and the approximate duration. The diary was collected at each follow-up visit and used for qualitative description and to assist in interpreting trends in adherence scale scores.

(4) Objective functional measures

All objective measurements were performed by a single trained rehabilitation therapist at each follow-up visit. ① Range of motion: Active wrist extension, flexion, radial deviation, and ulnar deviation angles of the affected side were measured using a universal goniometer. ② Grip strength: Grip strength of the affected side was measured using a calibrated Jamar dynamometer, with three consecutive measurements taken and the average value recorded.

### 2.3 Rehabilitation protocol

A fully standardized postoperative rehabilitation protocol was implemented uniformly across both study centers, with all patients receiving the same progressive rehabilitation guidance. The protocol was developed by the research team (including hand surgeons, rehabilitation therapists, and nursing staff) based on the literature and clinical consensus, and was divided into four phases.

Phase 1 (postoperative weeks 0–2): Active flexion and extension exercises of the affected fingers were initiated on postoperative day 1 (5 min per session, 3 times daily) to promote edema resolution and prevent tendon adhesions. Before suture removal at postoperative week 2, no active wrist motion was performed; only isometric contraction exercises of the forearm muscles were conducted (each contraction held for 5 s, followed by 5 s of relaxation, 10 repetitions per set, 3 sets daily).

Phase 2 (postoperative weeks 2–4): After suture removal, active wrist range-of-motion training was started, including wrist extension, flexion, radial deviation, and ulnar deviation. For each direction, the patient slowly moved the wrist to the maximum angle within the pain-free range, held the position for 5 s, and then relaxed. Each direction was repeated 10 times per set, with 3 sets completed daily. Patients were also encouraged to use the affected hand to assist in daily activities.

Phase 3 (postoperative weeks 4–8): Resistance training was added while maintaining joint range-of-motion exercises. Elastic bands were used for resisted wrist extension and flexion (resistance was set at a level that allowed 10 repetitions without causing significant pain), with 10 repetitions per set and 2 sets daily. Grip strength training was also initiated (squeezing a soft ball, holding each squeeze for 5 s, 10 repetitions per set, 3 sets daily). Patients were encouraged to gradually increase the load on the affected hand during daily activities (e.g., carrying a water cup, wringing a towel).

Phase 4 (postoperative weeks 8–12): Range-of-motion and strength training continued, with gradual increases in elastic band resistance and the number of grip strength repetitions according to patient tolerance. Functional task training was introduced, such as simulating unscrewing a bottle cap, lifting light objects (0.5–1 kg), and writing. Patients were encouraged to resume most activities of daily living, but lifting objects heavier than 2 kg or engaging in strenuous sports was avoided.

All patients received one-on-one rehabilitation instruction before discharge, including a printed illustrated manual and live demonstration. During hospitalization, training was supervised daily by a rehabilitation therapist. After discharge, patients were required to complete a daily home exercise diary (recording completion status and pain level) and submit it at follow-up visits at postoperative weeks 2, 4, 8, and 12. The research team reviewed the diary at each follow-up and provided corrective guidance for those whose training was not performed correctly. In addition, a telephone follow-up was arranged midway between visits (i.e., at postoperative weeks 6 and 10) to monitor progress and answer questions. The rehabilitation protocol was identical at both institutions, and all therapists received unified training and certification from the same training team.

### 2.4 Data collection and quality control

Standardized procedures were adopted for data collection in this study to ensure data quality. All researchers involved in data collection received uniform training prior to the start of the study. The training covered: detailed explanation of the study protocol and procedures, standardized instructions and completion guidelines for each scale, and standardized measurement methods for wrist range of motion and grip strength (using goniometers and dynamometers of the same brand and model, with operational techniques demonstrated and calibrated by a single senior therapist). Data collection was conducted in a dedicated assessment room at the outpatient clinic at four fixed follow-up time points (weeks 2, 4, 8, and 12), in a quiet and private environment. Each assessment followed a fixed sequence: first, patients independently completed the PRWE and the exercise adherence scale; researchers provided neutral explanations only when patients raised conceptual questions, without any suggestive guidance. Subsequently, objective functional measurements were performed by a designated assessor. To ensure response quality, researchers promptly checked the completeness of the scales on site after patients finished filling them out; any missing items or obviously contradictory responses were kindly pointed out and patients were asked to review and revise. All paper-based data were assigned unique identification codes within 24 hours of collection, and double-entered independently by two researchers into an encrypted electronic database. Consistency checks and error correction were performed using software comparison. In addition, a small-scale pilot study involving 30 patients was conducted prior to formal initiation to test the feasibility of the entire procedure, estimate scale reliability (reporting Cronbach's α coefficients), and refine operational details. The entire study strictly adhered to ethical guidelines: only information directly relevant to the study was collected, all data were anonymized, and regular internal quality audits were conducted by the research team.

### 2.5 Statistical analysis

All statistical analyses were performed using SPSS software (version 26.0) and R software (version 4.3.2). Continuous variables were described according to their distribution: normally distributed variables were presented as mean ± standard deviation, while non-normally distributed variables were presented as median and interquartile range. Categorical variables were described using frequencies and percentages. To test the primary research hypothesis, a linear mixed-effects model was employed. This model effectively handles within-subject correlations inherent in repeated-measures data. The total PRWE score served as the dependent variable. Fixed effects included measurement time, total exercise adherence score, and the key interaction term between time and adherence, while controlling for baseline covariates such as age, sex, and fracture classification. Random effects included a random intercept for each patient to account for inter-individual variability in initial status. The core focus of the model was to test the statistical significance of the interaction term, thereby determining whether exercise adherence moderated the slope of the functional recovery trajectory. Model goodness of fit was evaluated using the likelihood ratio test, Akaike Information Criterion (AIC), and Bayesian Information Criterion (BIC). To assess the consistency between patient-reported data and objective measurements, Pearson or Spearman correlation coefficients were calculated. For missing data that may occur in longitudinal studies, the primary analysis employed the linear mixed-effects model based on full information maximum likelihood estimation, which is an effective method for handling data missing at random. In addition, baseline characteristics were compared between participants who completed all follow-ups and those who dropped out to evaluate potential attrition bias. All statistical tests were two-sided, and *P* < 0.05 was considered statistically significant.

## 3 Results

### 3.1 Participant flow and baseline characteristics

**Figure 1:**
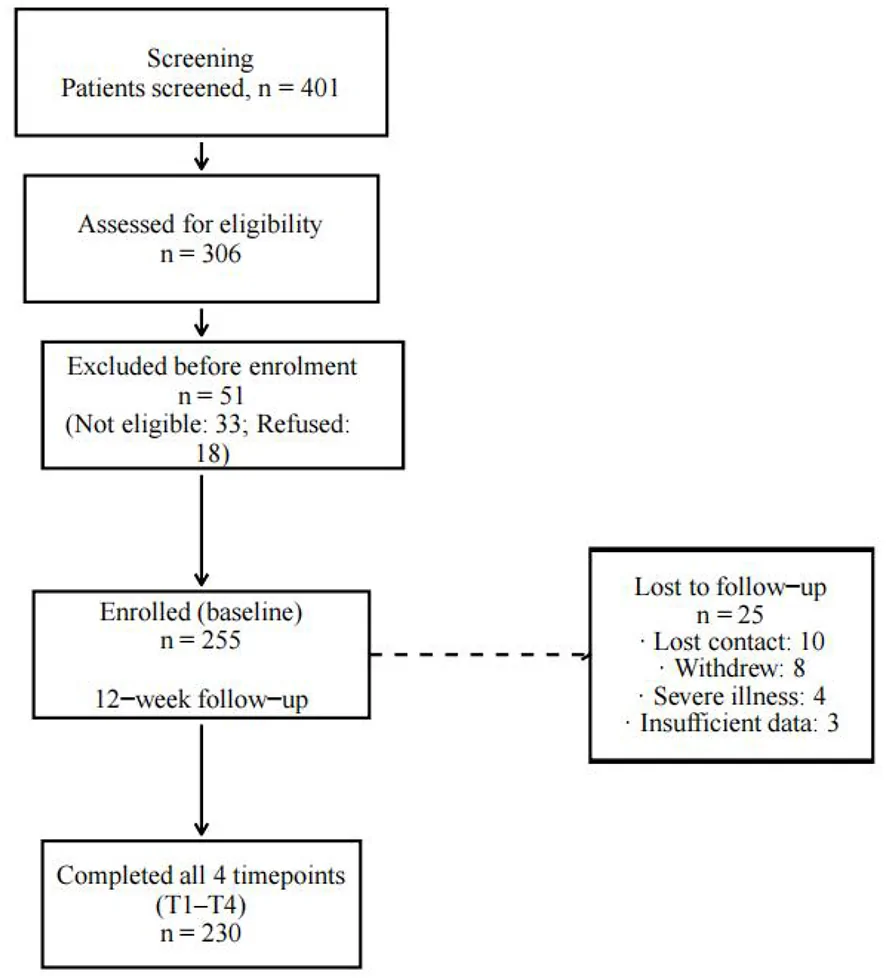
Flowchart of participant enrollment and follow-up.

The screening, enrollment, and follow-up procedures for study participants are presented in Figure 1. A total of 401 older patients who underwent volar locking plate internal fixation for distal radius fractures were screened across the two study centers. After evaluation, 306 patients met the preliminary inclusion criteria. Of these, 51 patients were excluded due to failure to meet specific inclusion criteria or refusal to participate. Ultimately, 255 patients provided written informed consent and entered the baseline assessment.

During the 12-week longitudinal follow-up, a total of 25 patients were lost to follow-up, yielding an overall dropout rate of 9.8%. The main reasons for dropout were as follows: 10 patients lost contact due to personal reasons (e.g., relocation, family caregiving); 8 patients explicitly declined to continue completing questionnaires during subsequent follow-ups; 4 patients withdrew due to other serious illnesses unrelated to the fracture (e.g., cardiovascular events); and 3 patients had excessive missing data due to non-compliance with the follow-up schedule. Ultimately, 230 patients completed assessments at all four time points, and their data were included in the final analysis. To assess whether loss to follow-up introduced selection bias, we compared the baseline characteristics between the 230 patients who completed all follow-up visits and the 25 patients who dropped out. No statistically significant differences were found between the two groups in terms of age, sex, body mass index, AO fracture classification, proportion of dominant hand injury, or prevalence of comorbid diabetes or osteoporosis (all *P* > 0.05). These results indicate that loss to follow-up in this study occurred largely at random, did not significantly affect the representativeness of the study sample, and therefore the subsequent analytical results are unlikely to be substantially influenced by attrition bias.

Based on the median baseline total exercise adherence score (60 points), the 230 patients were divided into a high-adherence group (*n* = 118) and a low-adherence group (*n* = 112). The baseline characteristics of the two groups are compared in Table 1. The results showed no statistically significant differences between the two groups in terms of age, sex, body mass index, AO fracture classification, proportion of dominant hand injury, or baseline kinesiophobia level (TSK-11 score) (all *P* > 0.05). Show in Table 1.

**Table 1:** Baseline characteristics of the high-adherence and low-adherence groups.

Characteristic	High-adherence group (*n* = 118)	Low-adherence group (*n* = 112)	Statistic	*P*-value
Age (years), M ± SD	68.0 ± 5.6	68.4 ± 5.8	*t* = −0.54	0.590
Sex, *n* (%)			χ^2^ = 0.23	0.631
Female	94 (79.7)	88 (78.6)		
Male	24 (20.3)	24 (21.4)		
BMI (kg/m^2^), M ± SD	24.0 ± 3.1	24.2 ± 3.3	*t* = −0.46	0.646
AO fracture classification, *n* (%)			χ^2^ = 1.12	0.571
Type A	42 (35.6)	43 (38.4)		
Type B	18 (15.3)	14 (12.5)		
Type C	58 (49.2)	55 (49.1)		
Dominant hand injured, *n* (%)	50 (42.4)	51 (45.5)	χ^2^ = 0.23	0.632
Baseline TSK-11 score, M ± SD	23.8 ± 6.5	25.2 ± 7.0	*t* = −1.58	0.116

BMI, body mass index; TSK-11, Tampa scale of kinesiophobia (11-item Chinese version); M ± SD, mean ± standard deviation.

### 3.2 Descriptive results and trends of major variables

**Figure 2:**
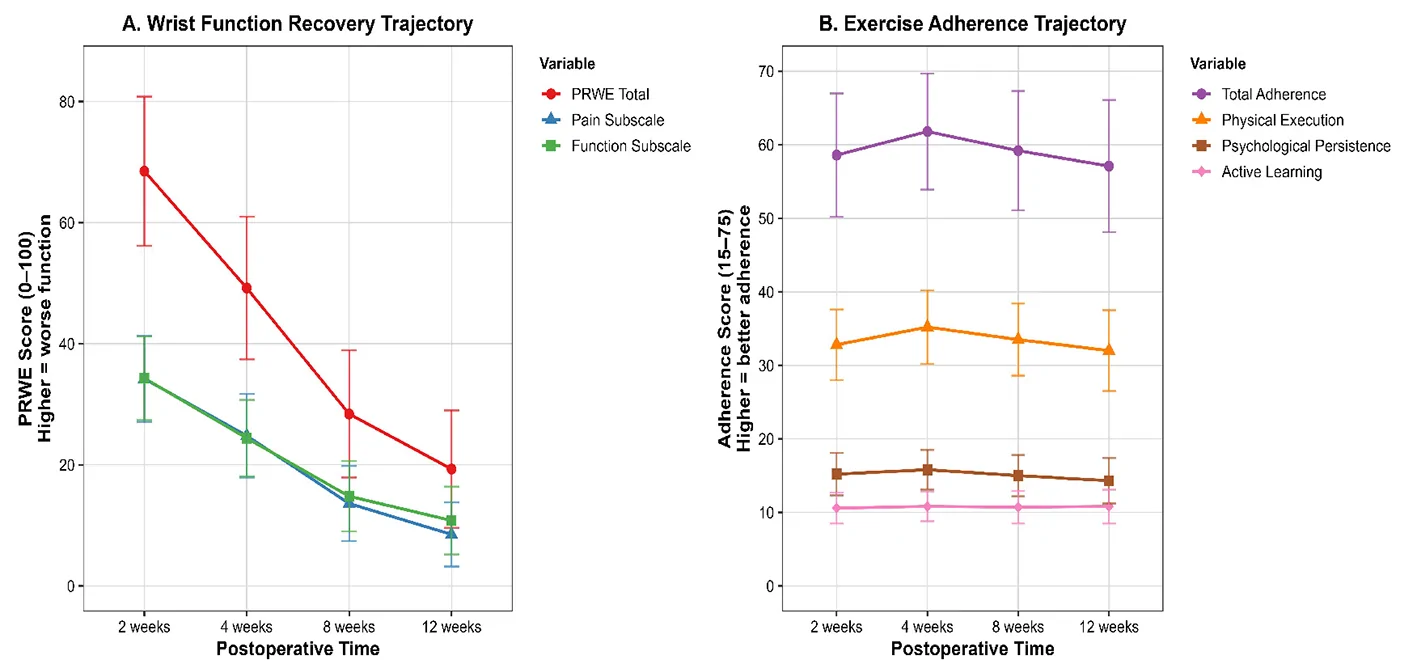
Trajectories of wrist function and exercise adherencein olderly patients with distal radius fracture (*n* = 230).

The measurements of each variable over the 12-week postoperative period are shown in Table 2 and Figure 2. Wrist function showed a continuous improvement trend, with the total PRWE score and its pain and function subscale scores decreasing significantly over time (main effect of time, all *P* < 0.001), with pain symptoms resolving earlier and more rapidly. The total exercise adherence score increased in the early postoperative period (up to week 4), peaked, and then gradually declined, with a significant main effect of time (*P* < 0.001). Among the dimensions, the physical execution dimension consistently had the highest adherence scores and showed a statistically significant change over time (*P* < 0.001); the psychological persistence dimension also changed significantly (*P* < 0.001); however, the active learning dimension scores did not differ significantly across time points (*P* = 0.308), suggesting that this dimension remained relatively stable.

**Table 2:** Descriptive statistics of major variables at each time point (*n* = 230).

Variable	Week 2	Week 4	Week 8	Week 12	F-value	*P*-value
PRWE total score	68.5 ± 12.3	49.2 ± 11.8	28.4 ± 10.5	19.3 ± 9.7	186.4	< 0.001
Pain subscale score	34.2 ± 7.1	24.8 ± 6.9	13.6 ± 6.2	8.5 ± 5.3	142.8	< 0.001
Function subscale score	34.3 ± 6.9	24.4 ± 6.3	14.8 ± 5.8	10.8 ± 5.6	168.2	< 0.001
Total exercise adherence score	58.6 ± 8.4	61.8 ± 7.9	59.2 ± 8.1	57.1 ± 9.0	21.3	< 0.001
Physical execution dimension	32.8 ± 4.8	35.2 ± 5.0	33.5 ± 4.9	32.0 ± 5.5	18.6	< 0.001
Psychological persistence dimension	15.2 ± 2.9	15.8 ± 2.7	15.0 ± 2.8	14.3 ± 3.1	9.7	< 0.001
Active learning dimension	10.6 ± 2.1	10.8 ± 2.0	10.7 ± 2.2	10.8 ± 2.3	1.2	0.308

Data are presented as mean ± standard deviation. PRWE total score ranges from 0 to 100, with higher scores indicating worse function. Total exercise adherence score ranges from 15 to 75, with higher scores indicating better adherence. F-values and *P*-values are from the main effect of time in repeated-measures ANOVA (Greenhouse–Geisser correction applied when sphericity assumption was violated).

### 3.3 Interactive relationship between functional recovery trajectory and exercise adherence

To examine the dynamic interactive relationship between wrist function recovery trajectory and exercise adherence, a linear mixed-effects model was constructed. The model used total PRWE score as the dependent variable, with measurement time (continuous variable, in weeks, coded as 2, 4, 8, 12), total exercise adherence score (time-varying covariate, entered as raw score), and their interaction term as core fixed effects. Covariates included age, sex, AO fracture classification (with type A as reference), dominant hand injury status, and baseline kinesiophobia level (TSK-11 score). Random effects included individual intercepts to account for between-patient variability in initial functional status. Model fitting results are detailed in Table 3.

**Table 3:** Parameter estimates of the linear mixed-effects model predicting total PRWE score.

Fixed effects	Estimate	SE	*t* value	*P* value	95% CI
Intercept	75.32	4.56	16.52	< 0.001	66.38, 84.26
Time (week)	−6.82	0.31	−22.00	< 0.001	−7.43, −6.21
Total exercise adherence	−0.53	0.12	−4.42	< 0.001	−0.77, −0.29
Time × adherence	−0.04	0.02	−2.03	0.043	−0.08, −0.001
Age	0.12	0.08	1.50	0.135	−0.04, 0.27
Sex (female vs. male)	2.15	1.62	1.33	0.185	−1.03, 5.33
AO type B vs. A	1.89	1.98	0.95	0.342	−1.99, 5.77
AO type C vs. A	3.74	1.73	2.16	0.032	0.35, 7.13
Dominant hand injured (Yes vs. No)	2.83	1.39	2.04	0.042	0.11, 5.55
Baseline TSK-11 score	0.64	0.15	4.27	< 0.001	0.35, 0.94
**Random effects**	**Variance component**		**SE**	**Wald Z**	***P*** **value**
Between-subject intercept	18.54		2.73	6.78	< 0.001
Residual	29.63		1.71	17.33	< 0.001

PRWE total score ranges from 0 to 100, with higher scores indicating worse function. Total exercise adherence score ranges from 15 to 75, with higher scores indicating better adherence. The model adjusted for age, sex, AO fracture classification, dominant hand injury status, and baseline kinesiophobia level.

Fixed-effects analysis showed that the main effect of time was statistically significant (estimate = −6.82, SE = 0.31, *t* = −22.00, *P* < 0.001), indicating that, holding other factors constant, the total PRWE score decreased by an average of 6.82 points per additional week, reflecting significant improvement in wrist function over time. The main effect of exercise adherence was also significant (estimate = −0.53, SE = 0.12, *t* = −4.42, *P* < 0.001), indicating that at any given time point, each 1-point increase in exercise adherence was associated with an average 0.53-point decrease in total PRWE score, suggesting better functional status among patients with higher adherence.

The interaction term between time and exercise adherence was statistically significant (estimate = −0.04, standard error = 0.02, *t* = −2.03, *P* = 0.043). The *P*-value for this interaction effect was at the borderline level of significance (*P* = 0.043), and the effect size was modest (β = −0.04, 95% CI: −0.08 to −0.001). This negative interaction indicated that the facilitative effect of exercise adherence on functional recovery became progressively stronger over time. In other words, patients with higher adherence exhibited a significantly faster rate of decline in PRWE scores (i.e., faster functional recovery) compared to those with lower adherence.

**Figure 3:**
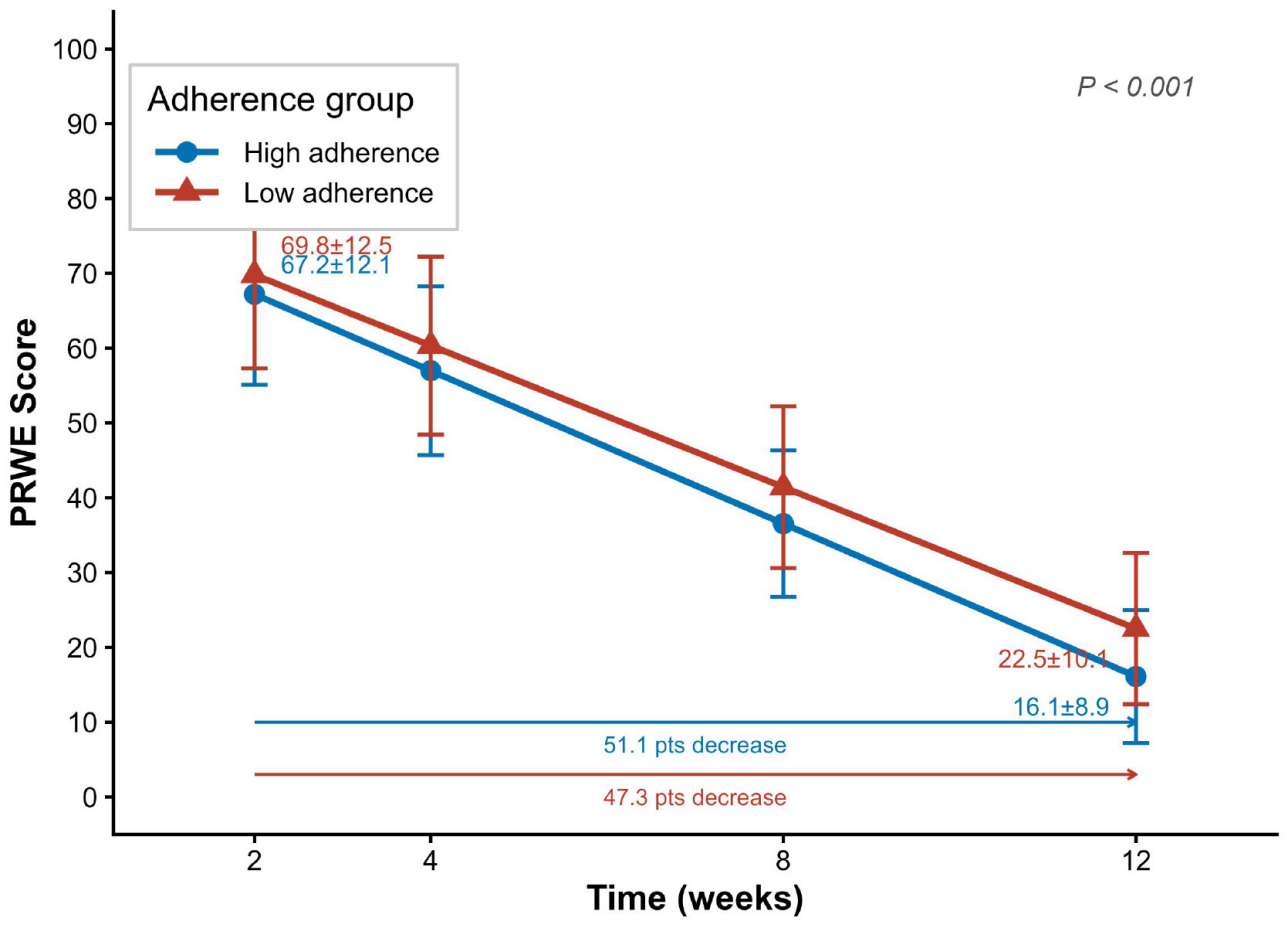
Recovery trajectories of wrist function by exercise adherence level.

To further illustrate the direction of the interaction, patients were divided into a high-adherence group (≥60 points, *n* = 118) and a low-adherence group (< 60 points, *n* = 112) based on the median baseline adherence score, and the trends of total PRWE scores over time were plotted for both groups. The results showed that at 2 weeks postoperatively, the difference in total PRWE scores between the two groups was small (high-adherence group: 67.2 ± 12.1 vs. low-adherence group: 69.8 ± 12.5, *P* = 0.108). By 12 weeks postoperatively, the high-adherence group had significantly lower total PRWE scores than the low-adherence group (16.1 ± 8.9 vs. 22.5 ± 10.1, *P* < 0.001). The difference between the two groups was 6.4 points. Moreover, the mean decrease from 2 to 12 weeks was 51.1 points in the high-adherence group, which was greater than the 47.3-point decrease observed in the low-adherence group (see Figure 3).

Regarding random effects, the variance component for the between-subject intercept was 18.54 (Wald Z = 6.78, *P* < 0.001), indicating significant inter-individual variability in initial functional status at 2 weeks postoperatively. The residual variance was 29.63, suggesting that the model explained a substantial proportion of the total variance. Model diagnostics showed that both the normality test of residuals (Shapiro–Wilk test, *P* = 0.213) and the homogeneity of variance test (Levene's test, *P* = 0.376) satisfied the assumptions of the linear mixed model.

### 3.4 Supplementary analyses

#### 3.4.1 Convergent validity

To evaluate the consistency between patient-reported outcome measures and objective functional measurements, Pearson correlation coefficients were calculated at each follow-up time point between the PRWE function subscale score, total exercise adherence score, and the objective measures of active wrist range of motion (total arc of extension and flexion) and grip strength. The results are presented in Table 4.

**Table 4:** Correlation coefficients between self-report scales and objective measures at each time point.

Variable pair	2 weeks postop	4 weeks postop	8 weeks postop	12 weeks postop
PRWE function vs. total wrist ROM	−0.48^***^	−0.56^***^	−0.65^***^	−0.71^***^
PRWE function vs. grip strength	−0.39^***^	−0.47^***^	−0.55^***^	−0.62^***^
Total exercise adherence vs. total wrist ROM	0.31^***^	0.38^***^	0.45^***^	0.49^***^
Total exercise adherence vs. grip strength	0.26^***^	0.33^***^	0.39^***^	0.44^***^

^***^P < 0.001. Total wrist range of motion is defined as the sum of extension and flexion angles.

At all time points, the PRWE function subscale score showed moderate to strong negative correlations with total wrist range of motion (*r* range: −0.48 to −0.71, all *P* < 0.001) and with grip strength (*r* range: −0.39 to −0.62, *P* < 0.001), indicating that greater self-reported functional impairment was associated with lower objective joint mobility and muscle strength. Total exercise adherence score showed significant positive correlations with concurrent total wrist range of motion (*r* = 0.31 to 0.49, *P* < 0.001) and grip strength (*r* = 0.26 to 0.44, *P* < 0.001), suggesting that patients with higher self-reported adherence objectively exhibited better functional status. The above results provide supporting evidence for the convergent consistency between the PRWE and exercise adherence scale and the objective functional measures. It should be noted that the full validation of a scale's validity requires systematic, multi-method psychometric procedures, including the accumulation of multidimensional evidence such as content validity, construct validity, and criterion-related validity. Correlation coefficients observed in a single study can serve as a component of validity evidence but are insufficient to independently establish the validity of a scale.

#### 3.4.2 Model comparison and goodness of fit

To evaluate whether the inclusion of the interaction term significantly improved model fit, a likelihood ratio test (LRT) was conducted comparing two nested models: Model 0 (without the time × adherence interaction term) and Model 1 (with the interaction term). The Akaike Information Criterion (AIC) and Bayesian Information Criterion (BIC) were also reported as supplementary indices of goodness of fit. The results are presented in Table 5.

**Table 5:** Comparison of nested models.

Model	−2LL	AIC	BIC	Comparison	χ^2^	df	*P* value
Model 0 (without interaction term)	2448.9	2458.9	2500.4	—	—	—	—
Model 1 (with time × adherence)	2444.8	2456.8	2498.3	Model 1 vs. Model 0	4.12	1	0.042

−2LL, −2 log-likelihood; AIC, Akaike Information Criterion; BIC, Bayesian Information Criterion. Both models included the same random effects structure (random intercept) and covariates (age, sex, AO fracture classification, dominant hand injury, and baseline TSK-11 score).

The likelihood ratio test showed that the −2 log-likelihood (−2LL) value of Model 1 was significantly lower than that of Model 0 (χ^2^ = 4.12, df = 1, *P* = 0.042), indicating that the addition of the interaction term resulted in a significant improvement in model fit. The AIC (2456.8) and BIC (2498.3) of Model 1 were both lower than those of Model 0 (AIC = 2458.9; BIC = 2500.4), further supporting that the model retaining the interaction term was more appropriate. These findings are consistent with the significance of the interaction coefficient shown in Table 3, collectively confirming the moderating effect of exercise adherence on the functional recovery trajectory.

#### 3.4.3 Model diagnostics and assumption testing

To verify the appropriateness of the linear mixed-effects model, the following supplementary analyses were conducted.

(1) Time function form test: Given that early postoperative functional recovery may follow a nonlinear trend, three time specifications were compared: ① a linear model (time as a continuous variable); ② a quadratic model (with a time^2^ term); and ③ a categorical time model (time as a four-level dummy variable). The likelihood ratio test showed that the quadratic term was not significant (*P* = 0.18), and the AIC did not decrease (linear model AIC = 3247; quadratic model AIC = 3251). The marginal means of the categorical time model were highly consistent with the predicted values from the linear model (difference < 1.2 points), so the linear specification was retained.
(2) Random effects structure: The initial model included both a random intercept and a random slope (for time). The variance component of the random slope was close to zero, and the likelihood ratio test showed no significant improvement in model fit after adding the random slope (χ^2^ = 1.31, *P* = 0.25). Therefore, only the random intercept was retained in the final model.
(3) Multicollinearity diagnostics: The variance inflation factors (VIF) for all independent variables were less than 2.5, indicating no serious multicollinearity.
(4) Residual assumption verification: The scatterplot of residuals vs. fitted values suggested approximately homoscedasticity; the Q-Q plot of residuals approximated a straight line, and the Shapiro–Wilk test (*P* = 0.213) supported the normality assumption. See Table 6.

**Table 6:** Linear mixed-effects model diagnostics and assumption testing results.

Test item	Test content	Statistic/indicator	Result	Conclusion
Time function form	Linear vs. quadratic model	Likelihood ratio test	χ^2^ = 1.85, *P* = 0.18	Quadratic term not significant; linear model preferred
	Linear vs. categorical time model	AIC comparison	Linear AIC = 3247, Categorical AIC = 3252	Linear model more parsimonious; predicted values highly consistent
Random effects structure	Random intercept vs. random intercept + random slope	Likelihood ratio test	χ^2^ = 1.31, *P* = 0.25	Random slope not significant; retain random intercept
Multicollinearity	VIF for all independent variables	Variance inflation factor	Min = 1.12, Max = 2.43, all < 2.5	No serious multicollinearity
Residual normality	Shapiro–Wilk test	W statistic	W = 0.991, *P* = 0.213	Residuals approximately normal
Residual homoscedasticity	Levene's test	F statistic	F = 1.08, *P* = 0.376	Residuals homoscedastic

#### 3.4.4 Sensitivity analysis

To examine the robustness of the primary analytical results, two sensitivity analyses were conducted. First, the same linear mixed-effects model described in Section 3.3 was refitted using data from only the 230 patients who completed all four follow-up visits. Second, missing data were handled using multiple imputation (based on chained equations, m = 20 imputed datasets), and the interaction effect was analyzed using pooled estimates. The results are presented in Table 7. In the completer analysis, the interaction between time and exercise adherence remained statistically significant (estimate = −0.039, SE = 0.019, *t* = −2.05, *P* = 0.041), with direction and magnitude consistent with the primary analysis (interaction estimate = −0.040 in the primary analysis). The pooled analysis based on multiple imputation also showed a significant interaction effect (estimate = −0.041, SE = 0.020, *t* = −2.05, *P* = 0.040). Furthermore, the main effects of time and exercise adherence remained significant across all sensitivity analyses. These findings indicate that the moderating effect of exercise adherence on the functional recovery trajectory identified in this study was not substantially influenced by attrition bias or missing data handling methods, supporting the robustness of the conclusions.

**Table 7:** Parameter estimates of core fixed effects in sensitivity analyses.

Analytical model	Fixed effect	Estimate	SE	*t* value	*P* value
Primary analysis (full sample, FIML)	Time	−6.820	0.310	−22.00	< 0.001
	Exercise adherence	−0.530	0.120	−4.42	< 0.001
	Time × adherence	−0.040	0.020	−2.03	0.043
Sensitivity analysis 1 (completers, *n* = 230)	Time	−6.815	0.312	−21.84	< 0.001
	Exercise adherence	−0.529	0.121	−4.37	< 0.001
	Time × adherence	−0.039	0.019	−2.05	0.041
Sensitivity analysis 2 (multiple imputation, m = 20)	Time	−6.822	0.313	−21.79	< 0.001
	Exercise adherence	−0.532	0.122	−4.36	< 0.001
	Time × adherence	−0.041	0.020	−2.05	0.040

FIML, full information maximum likelihood. All models adjusted for age, sex, AO fracture classification, dominant hand injury status, and baseline kinesiophobia level.

## 4 Discussion

Through a 12-week longitudinal follow-up of 230 older patients after distal radius fracture surgery, the present study found that wrist function (total PRWE score) continuously improved over time, and that this recovery trajectory was significantly moderated by exercise adherence. The linear mixed-effects model revealed a statistically significant interaction between time and exercise adherence (β = −0.04, *P* = 0.043), indicating that patients with higher adherence exhibited a significantly faster rate of PRWE decline compared to those with lower adherence. In other words, patients who consistently adhered to rehabilitation exercises not only had better functional status at any given time point but also recovered at a faster rate. This “acceleration effect” was particularly pronounced during the mid-to-late postoperative period (8 to 12 weeks), with the PRWE gap between the high- and low-adherence groups widening from 2.6 points at 2 weeks postoperatively to 6.4 points at 12 weeks postoperatively.

Previous studies have also supported the positive association between exercise adherence and functional outcomes. A meta-analysis of patients following distal radius fracture surgery (13) found that those receiving early mobilization instructions achieved superior wrist range of motion and upper extremity function compared to those receiving conventional immobilization, and that adherence scores were positively correlated with functional recovery (25). MacDermid et al. (22), in their validation study of the PRWE, noted that patient-reported functional impairment was highly consistent with objective measures; however, the role of adherence in this relationship was not fully explored. Compared with these studies, the innovation of the present study lies in incorporating adherence as a time-varying covariate into a longitudinal model and directly testing the interaction effect between adherence and time, thereby revealing the moderating effect of adherence on recovery speed rather than merely a static correlational relationship.

The potential mechanisms underlying this phenomenon can be understood from several perspectives. From a physiological standpoint, regular joint activity promotes local blood circulation, reduces swelling, and prevents joint adhesion and muscle atrophy (26). These benefits are dose-dependent: the higher the adherence, the more adequately the tissues are stimulated, and the faster the repair process, which is consistent with the findings of Celeiro (27). From a psychological perspective, Bandura's social cognitive theory posits (28) that successful completion of exercise tasks enhances patients' self-efficacy, motivating them to engage more actively in subsequent rehabilitation, thereby forming a positive cycle of “exercise → functional improvement → enhanced confidence → more exercise,” consistent with the study by Stern (29). Conversely, patients with low adherence may avoid activity due to early pain or fear, leading to delayed functional recovery, which in turn reinforces the negative expectation that “movement causes pain,” trapping them in a vicious cycle of “fear–avoidance–disuse” (30). In the present study, the baseline kinesiophobia level was a significant positive predictor of total PRWE score (β = 0.64, *P* < 0.001), indirectly supporting this explanation.

In the present study, the total exercise adherence score showed an initial increase followed by a decline, rising from 58.6 points at 2 weeks postoperatively to 61.8 points at 4 weeks, and then decreasing to 59.2 points and 57.1 points at 8 and 12 weeks, respectively. This pattern suggests that older patients exhibit relatively strong rehabilitation motivation and execution ability in the early postoperative period (particularly from 2 to 4 weeks), but adherence attenuates over time. Similar phenomena have been reported in other orthopedic rehabilitation studies. Mahmood et al. (31), in their systematic review, reported that home exercise adherence tends to be high at the initial stage of treatment and gradually declines thereafter, with approximately 50% of patients failing to fully comply with prescribed regimens after 6 months. Jack et al. (32), in their systematic review of barriers to physiotherapy adherence, also identified lack of supervision, insufficient positive feedback, pain, and fatigue as major contributors to dropout from home-based exercises. The findings of the present study are consistent with the aforementioned literature, but by narrowing the observation window to 12 weeks, they more clearly delineate the inflection point of the “initial increase followed by decline” in adherence, which occurs between 4 and 8 weeks postoperatively. The reasons for the decline in adherence warrant in-depth analysis. First, at 4 weeks postoperatively, patients typically have their braces removed or immobilization reduced, and pain is significantly alleviated. At this stage, they may mistakenly believe that they have “fully recovered” and consequently relax their exercise intensity. Second, as function gradually improves, activities of daily living (such as eating and dressing) begin to assume part of the role of “passive exercise,” leading patients to underestimate the necessity of active training. Third, older patients often present with multiple comorbidities (e.g., diabetes mellitus, osteoporosis) or experience insufficient social support, such as transportation difficulties or lack of family supervision, making it challenging to maintain adherence in the mid-to-late phase. These findings suggest that clinicians should monitor adherence throughout the entire rehabilitation period and strengthen follow-up and motivational support during the critical window of 4 to 8 weeks postoperatively. Compared with previous studies, the uniqueness of the present study is reflected in the following aspects. First, patient population: This study specifically focused on older patients aged 60 years and above after distal radius fracture surgery, whereas most previous studies covered all age groups or predominantly included younger patients (33). Older adults have distinct characteristics in terms of osteoporosis background, comorbidity burden, kinesiophobia, and social support; therefore, their functional recovery trajectory and adherence patterns may differ fundamentally from those of younger patients (34). Second, rehabilitation strategy: This study adopted a standardized, phased, progressive rehabilitation protocol with clear specifications of the starting time, training content, intensity, and frequency for each phase, and the protocol was fully uniform across the two centers. In contrast, many previous studies only vaguely described “routine rehabilitation” or “early mobilization,” lacking reproducible operational details (1, 8). Third, follow-up duration and density: This study scheduled four intensive follow-up time points within the 12-week postoperative period, enabling detailed characterization of the dynamic functional recovery trajectory. Most previous studies employed only single or double assessments, making it difficult to capture nonlinear changes and inflection points during recovery (35). Fourth, adherence assessment approach: This study incorporated exercise adherence as a time-varying covariate into the longitudinal model, rather than treating it as a fixed baseline predictor. This analytical approach better reflects the real-world nature of adherence fluctuating over time and with functional status. In contrast, previous studies mostly relied on single retrospective self-reports or simple descriptions of adherence rates (31, 32). Fifth, statistical methodology: This study employed a linear mixed-effects model to directly test the interaction effect between time and adherence, thereby revealing the moderating role of adherence on the rate of recovery (rather than merely on endpoint status). Previous studies mostly used repeated-measures analysis of variance or simple correlation/regression, which cannot isolate the dynamic moderating effect of adherence (15, 16). Collectively, these differences highlight the methodological innovations of the present study and provide more nuanced evidence for understanding the role of adherence in geriatric fracture rehabilitation.

The convergent validity analysis of the present study showed that the PRWE function subscale was moderately to strongly negatively correlated with wrist range of motion and grip strength (*r* = −0.39 to −0.71), and the total exercise adherence score was positively correlated with objective functional measures (r = 0.26 to 0.49). The magnitude of these correlation coefficients was consistent with the range reported in the previous literature. MacDermid et al. (22) found that, in patients with distal radius fractures, the PRWE total score correlated with wrist range of motion (*r* = −0.52 to −0.68) and with grip strength (*r* = −0.41 to −0.60). The results of the present study are similar, indicating that the Chinese version of the PRWE also demonstrates good criterion-related validity in the older adult population. It should be clarified that the above comparison is intended to validate the outcome measurement instrument, rather than to directly support the primary study hypothesis (i.e., the association between exercise adherence and functional recovery trajectory). The satisfactory psychometric properties of the PRWE provide a methodological foundation for the reliable detection of adherence effects in the main analysis of this study. However, although the correlations between the total adherence score and objective measures were significant, they were relatively weak (*r* < 0.5), which may reflect the inherent social desirability bias and recall bias of self-reported adherence. Nevertheless, these moderate positive correlations still support the reliability of the adherence scale as a group-level assessment tool. Future studies may consider incorporating objective monitoring methods such as wearable devices or electronic diaries to further improve the accuracy of adherence measurement.

Although the interaction effect size between time and adherence appeared modest (β = −0.04), this effect represents additional weekly improvement. Over the cumulative 12-week period, the high-adherence group showed a greater decline of approximately (0.04 × 10 weeks × adherence difference) points compared with the low-adherence group, resulting in an actual between-group PRWE difference of 6.4 points. Although this difference is slightly below the reported minimal clinically important difference (MCID) for the PRWE of approximately 11.5 points (36), considering the unique characteristics of the older adult population in terms of functional baseline and recovery potential, a difference of 6.4 points may still carry clinical perceptibility at the individual level. Furthermore, at 12 weeks postoperatively, the mean PRWE scores were 16.1 points in the high-adherence group and 22.5 points in the low-adherence group. The high-adherence group had approached normal functional levels, whereas the low-adherence group remained within the range of mild functional impairment. This distinction holds reference value in clinical decision-making. This study incorporated exercise adherence as a time-varying covariate into a linear mixed-effects model, revealing a positive association between adherence and the functional recovery trajectory. However, it should be clearly noted that the directionality of this association remains uncertain. Although the present study tends to view adherence as a facilitator of functional improvement, reverse causality is equally plausible: patients who recover more quickly may be more motivated to persist with exercise due to reduced pain, increased confidence, and a clearer perception of rehabilitation benefits, thereby demonstrating higher adherence. In other words, exercise adherence may be both a cause and a consequence of functional improvement, with a potentially bidirectional reinforcing cycle between the two. This interpretation is consistent with Bandura's social cognitive theory (28), which emphasizes the reciprocal shaping relationship between self-efficacy and behavior. Given the observational design of this study, although the linear mixed-effects model can handle time-varying covariates, it cannot establish causality. Future studies may employ cross-lagged panel models or marginal structural models to further elucidate the directional relationship between adherence and functional recovery, and to explore the dynamic causal pathways between the two over time.

The findings of this study have important practical implications for postoperative rehabilitation management in older patients with distal radius fractures. First, clinicians should regard exercise adherence as a dynamic, modifiable therapeutic target rather than a static patient trait. In the early postoperative period (weeks 2 to 4), emphasis should be placed on providing clear written and verbal instructions, and helping patients establish correct exercise techniques through live demonstrations and corrective feedback. During the critical window period after adherence peaks (weeks 4 to 8), attention should be paid to the decline in adherence, and the following stratified maintenance strategies should be implemented: ① adding a telephone follow-up at postoperative week 6 to monitor training progress, address questions, and provide positive encouragement; ② recommending rehabilitation mobile applications for suitable patients to provide timed reminders, video demonstrations, and progress tracking functions, while offering simple paper-based exercise calendars assisted by family members for older patients who are not familiar with smart devices; and ③ encouraging family members to participate in the patient's daily exercise, such as accompanying practice, providing emotional support, or assisting with filling in the exercise diary. Second, baseline kinesiophobia level (TSK-11) can be used as a screening tool to identify patients with a high fear-avoidance tendency (e.g., TSK-11 ≥ 28), for whom additional cognitive-behavioral interventions should be provided, including one to two sessions of face-to-face psychological counseling or referral to a rehabilitation therapist for specialized guidance, in order to break the “fear-avoidance” cycle. For such high-risk patients, periodic supervised rehabilitation may also be considered to ensure exercise quality and safety. Third, given the bidirectional reinforcing relationship between adherence and functional recovery, rehabilitation programs should be designed to balance “immediate sense of achievement” with “long-term sustainability.” For example, setting short-term phased goals, such as “increase wrist extension by 5° this week” or “independently complete wringing a towel this week,” enables patients to see progress in a timely manner, thereby strengthening intrinsic motivation. At each follow-up visit, therapists should explicitly acknowledge the patient's effort and progress, rather than focusing solely on improvements in functional scores. In addition, the establishment of patient support groups or online communities may be considered to further enhance patients' rehabilitation confidence and persistence through peer support and experience sharing. The implementation of the above strategies should be individualized according to the patient's personal characteristics (age, cognitive function, social support, technological literacy) to maximize intervention effectiveness and resource utilization efficiency.

This study has several limitations that should be considered when interpreting the results. First, sample representativeness and generalizability. This study only included older patients from two hospitals in Nantong City, with the sample originating from the same geographic region, which may introduce selection bias and limit the generalizability of the findings to other regions, community hospitals, or advanced trauma centers. Furthermore, the conclusions of this study are applicable only to older patients who underwent volar locking plate internal fixation and are not applicable to those who did not undergo surgery or received conservative treatment. Future studies should adopt multi-regional, multi-level research designs and include patients with different treatment modalities to improve external validity. Second, measurement of exercise adherence. Exercise adherence was assessed using a self-report scale. Although supplemented by cross-validation with home exercise diaries and objective functional measures, it remains difficult to completely eliminate social desirability bias and recall bias. Patients may tend to report higher levels of adherence than their actual behavior, leading to an overestimation of true adherence. Future studies may consider incorporating objective monitoring methods such as wearable devices, electronic diaries, or direct observation by therapists to further improve the accuracy of adherence measurement. Third, limitations of the follow-up period. The follow-up period of this study was 12 weeks postoperatively. Although this captures the critical window of early functional recovery, it is insufficient to assess medium-term (6-month) or long-term (1-year) functional maintenance or the persistence of adherence differences. Whether the 6.4-point PRWE difference between the high- and low-adherence groups at 12 weeks will be maintained, widened, or narrowed over a longer period remains unclear. Future studies should extend the follow-up period to 6 months or more than 1 year. Fourth, unmeasured confounders. Several important factors that may simultaneously influence functional recovery and exercise adherence were not included in the model of this study, including: ① pain-related factors (pain intensity, type and dosage of analgesic medication use); ② rehabilitation service utilization (number of physical therapy sessions, whether professional rehabilitation guidance was received); ③ psychosocial factors (level of family support, education level, occupational status, depressive symptoms, cognitive function, frailty status); ④ and clinical factors (quality of surgical reduction, postoperative complications, occurrence of complex regional pain syndrome). These unmeasured confounders may lead to residual confounding and affect the accuracy of effect estimates. Future studies should prospectively collect the above information or employ methods such as propensity score matching or instrumental variable analysis to further control for confounding bias. Fifth, limitations of causal inference due to study design. This study adopted an observational design. Although the linear mixed-effects model can handle time-varying covariates, it cannot establish causality. Exercise adherence may be either a cause or a consequence of functional improvement, and a bidirectional reinforcing relationship may exist between the two. Future studies could employ cross-lagged panel models or marginal structural models to further clarify the directional relationship between adherence and functional recovery. Sixth, heterogeneity in surgical and rehabilitation protocols. This study did not standardize surgical details (such as surgical approach, plate brand, screw configuration) or postoperative immobilization protocols, nor did it record the time interval from fracture to surgery. Variations in surgical experience and preferences among different surgeons may introduce unmeasurable variability. In addition, there may be differences in the actual execution of postoperative rehabilitation across patients. Although this study provided standardized rehabilitation guidance, actual implementation in the home environment remains difficult to fully monitor. Future studies should consider standardizing these variables or recording them prospectively to allow for adjustment in statistical analyses.

## 5 Conclusion

Through this 12-week longitudinal investigation, the present study found that wrist function in older patients after distal radius fracture surgery showed continuous improvement, while exercise adherence exhibited an initial increase followed by a decline, peaking at 4 weeks postoperatively. The linear mixed-effects model revealed a significant interaction between time and exercise adherence, indicating that higher adherence was associated with faster functional recovery, and this association was more pronounced in the mid-to-late postoperative period. These findings suggest that exercise adherence is an important clinical marker and a potential intervention target influencing the functional recovery trajectory. Clinicians should strengthen follow-up and motivation during the critical window period of 4 to 8 weeks postoperatively, and provide targeted support for patients with high levels of kinesiophobia, in order to optimize rehabilitation outcomes. It should be noted that this study adopted an observational design and cannot establish causality. Future research, including randomized controlled trials or quasi-experimental studies, is needed to further verify whether intervention strategies aimed at improving exercise adherence can lead to definitive functional improvements.

## Data Availability

The original contributions presented in the study are included in the article/supplementary material, further inquiries can be directed to the corresponding author.
